# Evaluating the feasibility and acceptability of the Namaste Care program in long-term care settings in Canada

**DOI:** 10.1186/s40814-020-00575-4

**Published:** 2020-03-02

**Authors:** Sharon Kaasalainen, Paulette V. Hunter, Vanina Dal Bello-Haas, Lisa Dolovich, Katherine Froggatt, Thomas Hadjistavropoulos, Maureen Markle-Reid, Jenny Ploeg, Joyce Simard, Lehana Thabane, Jenny T. van der Steen, Ladislav Volicer

**Affiliations:** 1 School of Nursing, McMaster University, 1280 Main Street West, HSC 3H48C, Hamilton, ON L8S 3Z1 Canada; 2 Department of Family Medicine, McMaster University, 1280 Main Street West, 3H48C, Hamilton, ON L8N 3Z5 Canada; 3 St. Thomas More College, University of Saskatchewan, Saskatoon, Canada; 4 School of Rehabilitation Science, McMaster University, Hamilton, Canada; 5 Leslie Dan Faculty of Pharmacy, University of Toronto, Toronto, Canada; 6 International Observatory on End of Life Care, Lancaster, UK; 7 Department of Psychology, University of Regina, Regina, Canada; 8 Aging, Community and Health Research Unit, School of Nursing, McMaster Institute for Research on Aging/Collaborative for Health and Aging, McMaster University, 1280 Main Street West, HSc 3N25B, Hamilton, ON L8S 4K1 Canada; 9 Aging, Community and Health Research Unit, School of Nursing, McMaster University, 1280 Main Street West, HSc 3N25C, Hamilton, ON L8S 4K1 Canada; 10 Land O Lakes, USA; 11 Department of Clinical Epidemiology and Biostatistics, McMaster University, Hamilton, ON Canada; 12 Department of Public Health and Primary Care, Leiden University Medical Center, Leiden, The Netherlands; 13 School of Aging Studies, University of South Florida, Tampa, FL USA

**Keywords:** Palliative care, Long-term care, Dementia

## Abstract

**Background:**

Residents living and dying in long-term care (LTC) homes represent one of society’s most frail and marginalized populations of older adults, particularly those residents with advanced dementia who are often excluded from activities that promote quality of life in their last months of life. The purpose of this study is to evaluate the feasibility, acceptability, and effects of Namaste Care: an innovative program to improve end-of-life care for people with advanced dementia.

**Methods:**

This study used a mixed-method survey design to evaluate the Namaste Care program in two LTC homes in Canada. Pain, quality of life, and medication costs were assessed for 31 residents before and 6 months after they participated in Namaste Care. The program consisted of two 2-h sessions per day for 5 days per week. Namaste Care staff provided high sensory care to residents in a calm, therapeutic environment in a small group setting. Feasibility was assessed in terms of recruitment rate, number of sessions attended, retention rate, and any adverse events. Acceptability was assessed using qualitative interviews with staff and family.

**Results:**

The feasibility of Namaste Care was acceptable with a participation rate of 89%. However, participants received only 72% of the sessions delivered and only 78% stayed in the program for at least 3 months due to mortality. After attending Namaste Care, participants’ pain and quality of life improved and medication costs decreased. Family members and staff perceived the program to be beneficial, noting positive changes in residents. The majority of participants were very satisfied with the program, providing suggestions for ongoing engagement throughout the implementation process.

**Conclusions:**

These study findings support the implementation of the Namaste Care program in Canadian LTC homes to improve the quality of life for residents. However, further testing is needed on a larger scale.

## Background

As the Canadian population continues to age, more people will die in long-term care (LTC), with estimates as high as 52.3% of residents dying in their LTC home by the year 2020 [1, 2]. Within LTC, over 75% of residents have dementia, which creates additional challenges to providing effective end-of-life (EOL) care due to the related cognitive, communication, functional, and behavioral problems that arise [3, 4]. Dementia is a life-limiting condition, with expected life expectancy ranging from 3 to 10 years from the time of diagnosis [5, 6]. The Quality End of Life Care Coalition of Canada and the Canadian Hospice Palliative Care Association jointly endorsed a national framework for a palliative approach to care of persons with chronic, progressive, life-limiting illnesses such as dementia, which includes a seamless transition from chronic disease management to appropriate EOL planning and care [7]. Due to the progressive and variable nature of the disease, a dementia-specific palliative approach to care is critical to promote quality of life [8]. Additional challenges related to quality EOL care include inappropriate and ineffective interventions, poor symptom management, and widespread lack of knowledge about palliative and dementia care [9–14].

Efforts to promote quality of life for residents with dementia have been neglected, particularly for those with advanced dementia. Moreover, rates of pain in this vulnerable population can be as high as 83%, since pain is often under-recognized or and undertreated [15–17]. Potential pain behaviors in dementia, such as aggression and yelling, are often misinterpreted as stemming from states of delirium or the dementia itself—this results in overprescribing of antipsychotics and other potentially unnecessary medications [18]. Given the negative side effects and costs of these medications, there is a need for less harmful, non-pharmacological alternatives for treating pain and pain-related behaviors, such as massage, gentle touch, and music [18, 19].

Some residents with advanced dementia are at risk of being marginalized. “Silent residents” [20] experience multiple losses of abilities as dementia progresses. Because “they are too frail to be difficult; they have lost that strength” (p.21), neither their verbal communication nor non-verbal behavior makes their need apparent [21]. As such, silent residents’ [20] are often left alone in their rooms, spending up to 87% of their days doing nothing [22] and less than 10 min per day engaged in some form of meaningful activity [23].

A new program, called Namaste Care, has great potential to improve quality of life, dignity, and comfort for LTC residents by engaging them in meaningful and therapeutic activities throughout the later stages of the disease trajectory [24–27]. Emerging evidence supports Namaste Care, with research showing reductions in antipsychotic and hypnotic use and behavioral symptoms [27–31]. The Namaste Care intervention is guided by concepts of person-centered care and maintaining a sense of “personhood” within a palliative approach to dementia care [27, 32–34]. It is intended for those individuals with moderate to advanced dementia and uses a gentle sensory stimulation and touch approach. The Namaste Care intervention should be implemented every day for 4 h each day by two LTC staff. Previous work involved an assessment of the barriers and facilitators to implementing Namaste Care [35] and evaluation of a training program to help launch its implementation [36]. The purpose of this study was to evaluate the feasibility and acceptability of the Namaste Care intervention and to understand the preliminary effects on quality of life, pain, mood, agitation, medication use, and medication costs for people with dementia living in LTC.

### Research questions

#### Primary

1. What are the feasibility, fidelity, and acceptability of the Namaste Care intervention in Canadian LTC?

#### Secondary

2. What is the potential effect of the Namaste Care intervention on quality of life, pain, medication use, and medication costs in residents with moderate to end-stage dementia over 6 months?

## Methods

### Design

This study used a mixed-method approach which combined both quantitative and qualitative methods to address the various research questions that, in combination, addressed the same overriding study goal [37]. Since the primary goal of this study was to evaluate the feasibility and acceptability of the Namaste Care intervention when it was implemented under real-world conditions that included reliance on usual care providers, a prospective one group, pre-post test design was used with no new staff hired to implement the intervention [38, 39].

A qualitative descriptive design was used for the post-implementation component (acceptability of the intervention), to explore perceptions about the intervention, to retrospectively examine perceived effects, barriers and facilitators to implementation, strategies to improve implementation, and sustainability of the intervention. Ethical approval for the study was approved by two universities (McMaster University: #2865; University of Saskatchewan Behavioural Research Ethics Board #15-267).

### Settings and participants

This intervention study was conducted in two, not-for-profit LTC homes: one in southern Ontario, a 127-bed facility (site 1), and a second one in Saskatchewan, a 60-bed facility (site 2). Both homes had a high proportion of residents with moderate to severe dementia and strong leadership investment in this project. Inclusion criteria for residents were as follows: over the age of 65, a diagnosis of dementia, English speaking, and a score of less than 40% on the Palliative Performance Scale (PPS) [40]. Written informed consent was obtained from each participant or their proxy.

### Namaste Care intervention

Based on initial feedback from the LTC staff at our study sites [35], the Namaste Care intervention for our study ran 5 days per week, 4 h per day for the first year of implementation. We used dedicated rooms for Namaste Care that were quiet and offered a high sensory, yet calming environment (e.g., soothing music, pleasant scents, soft lighting, warm blankets). Family members were encouraged to participate in preferred intervention activities (e.g., applying hand cream, brushing hair, assisting with nourishments), which previously has been viewed positively by residents and their family members [34]. Each session was facilitated by a trained carer.

#### Morning session

Before the residents entered the room, the Namaste Care atmosphere was already established. The room had moderate lighting and soft music and was at an appropriate temperature. Nursing assistants and home care staff transported residents to the room where they were greeted by the carer with a very welcoming and personal reception. Those staff that transported the residents were thanked for bringing the residents to the room. Residents were then reclined in their chairs to a comfortable position and given a blanket if the room was cool. Stuffed animals and realistic dolls were also offered to comfort the residents; some residents had specific animals and dolls that comforted them personally [33]. To engage the residents in meaningful activities, morning care was provided in the form of brushing hair, applying moisturizing lotion to hands and faces, giving massages, and providing nourishment and hydration throughout the session. Music therapists and volunteers provided stimulating music and musical activities including playing drums and other instruments. Once the session was over, staff returned the residents to their floors for lunch.

#### Afternoon session

The residents returned to the Namaste Care room for the afternoon session. The Namaste Care room reopened with the same calming environment as described above, with soft lighting, soothing music, and an appropriate temperature. Similar activities were performed in the afternoon session with the addition of snacks being offered to residents. The afternoon sessions concluded similarly to the morning sessions by the lights turned up and livelier music played over the speakers.

### Implementation strategy

A multifaceted approach was used to implement the Namaste Care intervention. We recruited carers at each home who were currently employed as personal support workers/care aides or activity aides. Two facility-wide education events were held, led by Joyce Simard, founder of Namaste Care [33]. Individualized training sessions with the carers were also held, to familiarize them with the equipment, describe the Namaste Care processes, and answer any questions. These sessions helped to launch the project and provide training to all staff, so that they were familiar with the program and able to spend time or fill in for a carer who was unable to work. A family meeting was conducted to introduce the program to the families of residents at each of the LTC sites. The research assistants and project leads (SK, PH) conducted outreach visits to each home on a weekly basis with the carers and other staff to assist with the implementation of the program, offer ongoing coaching, and enhance the fidelity of the intervention. As reminders, the project leaders and research assistants provided updates about the study to staff through the creation of posters and newsletters that were disseminated in the homes as well as during monthly site meetings to encourage staff to implement the Namaste Care intervention consistently. At monthly intervals, audit and feedback mechanisms were used to improve the fidelity of the intervention. This was accomplished by reviewing the Activity Checklists that were completed by the carers and by providing feedback to them about successes and areas for improvement.

### Procedure and outcomes

Research assistants worked with LTC staff to recruit residents that met the eligibility criteria. Once the staff received approval from the resident or proxy to be contacted by research staff, a research assistant met with the resident/proxy to explain the study and obtain written consent.

During the first 6 months of the program, there was continual enrollment of participants to maintain full capacity (i.e., when a participating resident passed away, the spot was filled with a new participant). In this manner, each resident received 6 months of the intervention except for dropouts (e.g., deaths), but the intervention period ran for an 18-month period. Measurement times for each resident were as follows: time 1 (pre-intervention), time 2 (3 months after time 1), and time 3 (6 months after time 1). We included a 3-month measurement time to accommodate dropouts due to death in our final analysis. That is, we used the 3-month assessments for those who died before the 6-month measurement time in our final analysis.

#### Feasibility of the intervention

Feasibility of the intervention relates to the degree to which the participants enroll in, complete, and comply with the intervention [38, 41]. The feasibility was monitored with the research activity log to assess the following pre-set target criteria: (a) reach of the intervention or proportion of intended target population that actually participates and reasons for non-participation (target: at least 80% consent who are eligible), (b) dose delivered or percentage of session delivered (target: at least 80% or 8/10 sessions attended per week) and duration of sessions (target: > 1.5 h per session), (c) retention rate (target: 90% have at least 3 months and 80% have at least 6 months of data collection completed), and (d) safety or adverse events reported (target: 0%). During instances when the program was halted due to infection outbreaks at the site, we removed these weeks from our calculations. However, if a resident did not attend Namaste Care due to an adverse event (i.e., skin breakdown), we included those sessions as missed, which had an impact on our overall feasibility rates.

#### Fidelity of the intervention

To assess intervention fidelity (extent to which the intervention is delivered as intended), the carers completed an activity checklist for each session/resident based on the core components of Namaste Care. The fidelity assessments included percentage of residents awake during the sessions, listening to music, having their hands and face washed, having a massage, drinking a beverage, having their hair brushed, having visits from family, and having their feet washed at each session that they attended.

#### Potential effect of the Namaste Care intervention

Once enrolled, the research assistant asked a staff person who worked closely with the resident to complete the outcome measures at baseline, 3 months, and 6 months post-implementation. The primary outcome was quality of life, which was measured by the QUALID [42, 43]. The QUALID is a proxy-report instrument that measures 11 observable behaviors indicating activity and emotional states, with higher scores indicating lower quality of life [43]. Each item is rated on a 5-point Likert scale, and the entire tool takes only 5 min to complete. Internal consistency was reported as good to excellent (Cronbach’s alpha = 0.77), and inter-item correlations were positive (ranged 0.17 to 0.70). Secondary outcomes included pain, medication use, and medication costs. Resident pain was assessed using the PACSLAC-II [44] using a standardized procedure that has been used in other evaluation studies and accommodates residents who have cognitive impairments [45]. Psychometric properties of the PACSLAC-II are very good with excellent validity [46]. Medication use was measured using the Medication Quantification Index (MQS) [47]. Scores were calculated for medications by considering the dosage level and the pharmacological class of the medication (e.g., narcotic analgesic, non-steroidal anti-inflammatory, antipsychotic, benzodiazepine). Medication costs were retrieved using the lowest cost interchangeable from the Ontario Drug Benefit (https://www.formulary.health.gov.on.ca/formulary/) and the Saskatchewan Online Formulary Database (http://formulary.drugplan.health.gov.sk.ca/). Medication usage was costed for the first month before implementing Namaste Care (baseline) and the first month (follow-up) after the intervention. Vitamins, supplements, other natural health products (NHPs), and temporary medications (i.e., antibiotics and immunizations) were excluded. Other demographic data was also collected (e.g., age, gender, diagnosis). Charlson Comorbidity Index (a measure that aims to categorize comorbid medical conditions that can alter mortality risk) [48] was calculated for each resident based on diagnoses in each chart.

#### Acceptability of the intervention

Acceptability of the intervention refers to the residents participating in Namaste Care and their family members’, carers’, and other LTC staffs’ perception of the intervention’s appropriateness, benefits, and convenience of implementation [41]. The acceptability of Namaste Care was assessed using individual interviews with family (*n* = 10), LTC staff (*n* = 28), and volunteers (*n* = 6). These interviews assessed their perceptions about the Namaste Care intervention in terms of (a) the intervention itself, including benefits of Namaste Care; (b) barriers and facilitators to implementation; and (c) suggestions for refining the Namaste Care intervention using a semi-structured interview guide (interview guide available upon request). Research assistants also documented field notes during their weekly meetings with staff. During these meetings, they discussed how Namaste Care was working, challenges staff were experiencing, and ways that staff members were addressing these challenges.

### Analysis

Descriptive analyses of participants’ characteristics and feasibility of the intervention were expressed as mean (standard deviation [SD]) and median (minimum-maximum) for continuous variables and count (percent) for categorical variables (Table 1). The analysis of feasibility outcomes (recruitment rate, dose delivered, intervention fidelity, and safety) was based on descriptive statistics reported as estimates with confidence intervals. These were evaluated against the criteria set for feasibility (Table 2). Changes in outcomes from pre- to post-implementation were examined using paired *t* tests. For residents who died before the 6-month intervention, we used the most recent outcome measurements that were taken as the post-assessment score in our paired *t* test analysis. The results were reported with 95% confidence intervals and associated *p* values. All statistical analyses performed used SAS 9.2 [49].

**Table 1:** Sample characteristics at baseline, *N* = 31

Variable	Descriptive statistic
Age (years), mean (SD)	86.4 (9.3)
Gender, *n* (%)	
Male	4 (12.9)
Female	27 (87.1)
Marital status, *n* (%)	
Single	2 (6.7)
Married	8 (26.7)
Divorced	1 (3.3)
Widow	19 (63.3)
Length of stay (years), mean (SD)	3.5 (3.8)
Charlson Comorbidity Index (CCI), mean (SD)	4.3 (1.7)
Rheumatic or connective tissue disease, *n* (%)	22 (71.0)
Hypertension, *n* (%)	21 (67.7)
Depression, *n* (%)	11 (35.5)
Cerebrovascular disease, *n* (%)	7 (22.6)
Previous myocardial infarction, *n* (%)	6 (19.4)
Congestive heart failure, *n* (%)	5 (16.1)
Diabetes mellitus, *n* (%)	5 (16.1)
Peripheral vascular disease, *n* (%)	3 (9.7)
Warfarin use, *n* (%)	3 (9.7)
Skin ulcers, *n* (%)	3 (9.7)
Pulmonary disease, *n* (%)	2 (6.5)
Renal disease, *n* (%)	2 (6.5)

*SD* standard deviation

**Table 2:** Feasibility indicators

Indicator	Study result	Pre-set criteria for success of feasibility
Participation rate	88.6%	> 80% consent who are eligible
Percentage of sessions attended per week	71.8%	At least 80% (8/10/week)
Average length of session	1.95 h	> 1.5 h
Retention rate: 3 months	90.3% (three died before 3 months)	90% have at least 3 months of data collection completed
Safety	10% (*n* = 3) residents developed skin breakdown/ulcers	No major injuries or adverse events reported (0%)

For the acceptability component of the study, the qualitative software program Dedoose [50] was used to help organize and analyze the data. The data from the focus group, individual interviews, and field notes were analyzed in a qualitative manner using thematic content analysis [51, 52]. Thematic content analysis involves identifying, analyzing, and interpreting patterns of meaning or “themes” within qualitative data [52]. To do this, we labeled important concepts that emerged from the data, and then categorized and coded them. Two investigators analyzed data separately for all interviews to foster credibility and dependability. Data analysis was conducted in an iterative manner until consensus was reached. A number of methods were used to improve the credibility of the findings (i.e., data triangulation of data sources and investigators).

## Results

### Characteristics of the participants

The average age of participating residents was 86.4 (SD = 9.3) years. The majority were female (87.1%) and widowed (63.3%). Participants had lived in LTC for an average of 3.6 (SD = 3.8) years. The average Charlson Comorbidity Index (CCI) score was 4.3 (SD = 1.7), with the most prevalent conditions (besides dementia) being rheumatic or connective tissue disease (71.0%), hypertension (67.7%), and depression (35.5%).

For the acceptability interviews, the majority of staff (*n* = 28) and volunteers (*n* = 6) who were interviewed were female (94%) and the median age was between 45 and 54 years of age (*n* = 29). They had been working with LTC residents with dementia for an average of 14.8 (*n* = 30, SD = 11.2) years. Out of the ten family members interviewed, four were female and six were male. The median age within the sample of family members was between 55 and 64 (*n* = 10) years, and their loved one had been a resident in LTC for 2.9 (*n* = 10, SD = 2.2) years.

### Feasibility

**Fig. 1:**
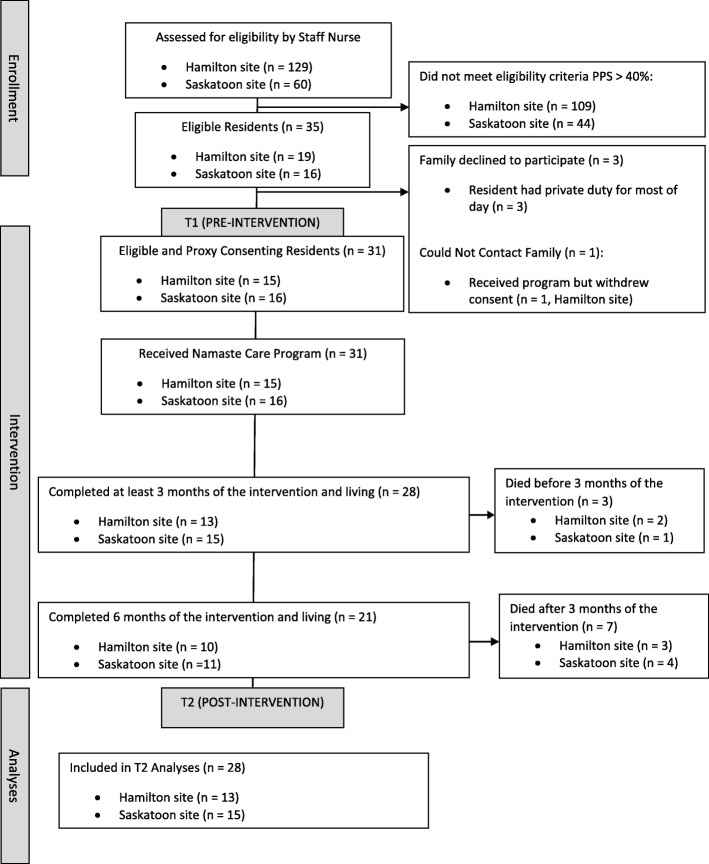
Intervention flow diagram

Based on the feasibility indicators that we determined before the study began, three of our five targets were reached including (a) 88.6% of residents who were eligible consented to participate or their proxies consented on their behalf (Fig. 1), (b) 90.3% completed at least 3 months of Namaste Care with data collected, and (c) the average length of session attended by residents was 1.95 h (see Table 2). The findings for the other two indicators that were not reached were (a) residents attended at least 71.8% of the sessions per week compared to expected target of 80% and (b) less than 10% of residents experienced an adverse event or injury (e.g., developed skin breakdown/ulcer) while attending Namaste Care while we expected no adverse events. However, there is no evidence that the skin breakdown was caused by the Namaste Care and would not happen without this intervention (Table 2).

### Fidelity

Intervention fidelity was also assessed in terms of the average percent of residents who received the components of Namaste Care at every session they attended (Table 3). The majority of residents were awake during the sessions (83.4%), listened to music (83.4%), had their hands (77.6%) and face (59.4%) washed, had a massage (66.9%), drank a beverage (61.9%), and had their hair brushed (54.5%) at each session that they attended. Program components that were implemented the most infrequently at each session included having visits from family (12.4%) and having their feet washed (11.0%) (Table 3).

**Table 3:** Intervention fidelity: mean percentage (%) of residents who received activity at each session

Program component	Mean^a^ (standard deviation)
Appears awake	83 (0.15)
Music	83 (0.15)
Massage	67 (0.13)
Drank beverage	62 (0.19)
Hair brushed	55 (0.11)
Range of motion	38 (0.12)
Scents	44 (0.16)
Pain assessment	24 (0.13)
Movie	18 (0.17)
Reading	18 (0.16)
Feet washed	11 (0.11)
Family visits	12 (0.14)

^a^Mean value was rounded to nearest whole number

### Effects of Namaste Care

The mean QUALID scores decreased somewhat following participation in the Namaste Care program suggesting a slight improvement in quality of life, but this was not statistically significant (*t* = 0.86, *p* = 0.40; see Table 4). A similar positive trend was also observed for pain (*t* = 0.82, *p* = 0.42). Further, medication use remained relatively constant (measured by the MQS). Of note, there was a statistically significant decrease in antidepressant use (*t* = 1.89, *p* = 0.05). Medication costs decreased slightly from baseline to post-intervention (Table 4).

**Table 4:** Outcome measures at baseline and 6 months, *N* = 28

Outcome measure	Baseline (T1) Mean (SD)	6 months (T2) Mean (SD)	Baseline to 6 months (T2–T1) mean difference (SD)	*t* (*p*)	95% confidence interval	
					Lower	Upper
Pain (PACSLAC-II)	6.0 (5.0)	5.3 (3.8)	− 0.7 (4.3)	0.82 (0.42)	− 0.97	2.26
Quality of life (QUALID)	26.4 (8.9)	24.7 (10.3)	− 1.7 (10.9)	0.86 (0.40)	− 2.35	5.77
Medication costs ($)	64.8 (58.1)	59.0 (52.5)	− 5.8 (53.6)	1.21 (0.24)	− 4.01	15.59
MQS benzodiazepine use	0.3 (1.0)	0.00 (0.0)	− 0.3 (1.0)	1.44 (0.16)	− 0.12	0.68
MQS antidepressant use	2.9 (3.3)	2.5 (3.3)	− 0.4 (3.3)	1.89 (0.05)	0.04	0.83
MQS antipsychotic use	1.4 (3.3)	1.4 (2.8)	0.0 (3.1)	0.00 (1.00)	− 0.76	0.76
MQS acetaminophen use	3.1 (2.3)	3.2 (2.5)	0.1 (2.5)	− 0.63 (0.53)	− 0.76	0.40
MQS NSAID use	1.2 (2.7)	1.1 (2.8)	− 0.1 (2.7)	0.44 (0.66)	− 0.44	0.69
MQS opioid use	2.1 (3.1)	2.8 (4.4)	0.7 (4.5)	− 1.07 (0.30)	− 2.19	0.69
MQS-III total score	15.8 (11.6)	15.1 (10.8)	− 0.7 (11.4)	0.69 (0.49)	− 1.36	2.74

*SD* standard deviation, *PACSLAC II* Pain Assessment Checklist for Seniors with Limited Ability to Communicate, range 0–31; *QUALID* Quality of Life in Late-Stage Dementia, range 11–55, higher scores represent lower QOL; *MQS* medication quantification scale; *NSAID* non-steroidal anti-inflammatory drug

### Acceptability

Qualitative findings from the interviews with families, staff, volunteers, and researcher field notes emerged across two main themes. First, staff and families described their experiences and many benefits of residents attending Namaste Care, including improved mood and engagement of residents and families feeling more empowered to interact with residents. Secondly, interview participants described some barriers and related recommendations to improve the implementation of Namaste Care (i.e., staffing the Namaste Care program, need for training for staff, managing adverse events such as skin breakdown). Each of these themes is described in more detail below.

#### Experiences and benefits of Namaste Care

Our study findings found positive experiences and benefits of Namaste Care as described by health care providers, volunteers, and family members. There are a number of “positive moments” where both family and staff described residents’ mood as improved during and after attending Namaste Care. During Namaste Care, participants described “in-the-moment” changes where Namaste Care seemed to “bring out the personality” of the residents, whereas before, staff and family felt residents were previously unaware of their surroundings. Some participants made comments that they felt residents were more alert in the Namaste Care room and engaged more with staff and family. A son of a resident stated:

Like there seems to be … a higher level of consciousness for the people that are here. Like they seem to be more able to recognize you, talk with you and you know even if they don’t talk but a gesture or whatever they can do. That level seems to be quite a bit higher with those people. I think it’s possibly because they are a little bit more satisfied (son)

The majority of participants interviewed described how the Namaste Care program appeared to improve residents’ mood. For example, a personal support worker/care aide stated:

I remember the care aides bringing in one resident … they said she was very sad, quiet, and very emotional that day. They weren’t sure if she should be there and I said, “sure bring her in … and I would say after lotioning and talking, and the music going and doing her hair and makeup, she started talking nonstop and she was laughing. By the end of it she was singing out loud with me. (PSW/CA)

Family members expressed similar comments, stating:

We all seem to agree that mom seems to be less anxious … .I had never seen mom smile like that -this huge big smile on her face. (daughter)

Another family member stated:

But mom enjoyed it and from observing other residents that were involved I could see where they really enjoyed it. Especially when you watched the smiles on their faces, and the enjoyment of the music. Some of them I had never seen them smile before or even had movement and all of a sudden they were tapping their toes and enjoying the music and doing a bit of exercises, and seemed very happy there (daughter)

A volunteer in the Namaste Care room commented on similar observations of residents. For example, one volunteer stated,

The change I’ve seen in her is that she will look you in the eye when you come in the room, and she will smile … I’ve just seen it continue from there. She laughs at us, and she has a smile. She sits with her eyes open and just a calm look on her face. She doesn’t smile all the time but she has her eyes open and she’s pleasant. (volunteer)

For some family members, the Namaste Care program had an impact on their views of the LTC home itself, where one participant describes how it helped them decide where to locate the resident:

Now that Dad’s gone we had talked about possibly moving her to another LTC home where we both live. But we know that, that’s [Namaste Care] is not available there and that’s why … that’s a big reason why I’m wanting to keep Mom here. Because we know that it’s a good program. (child)

Our study found that the majority of family interview participants expressed how participating in the Namaste Care program helped them feel more empowered and engaged in caring for residents who have advanced dementia. Some of the quotes from family members include:

Not like under pressure. I can come every other day and not feel bad, cause I know she has this going on. (daughter)

I can see that it’s very helpful. It’s something I would like to see continue. I think, wow, if I get to this point I would like something like this to be in place. I go away from here feeling such joy, you know? It’s not all about me but I’m getting the joy from just doing and helping. I can see that it’s making a difference. When you get a smile from somebody that is so sweet. (daughter)

I’ve only been doing it the 4 weeks, and I find that I usually get up excited in the morning to come, even the first day. I didn’t even know what to really expect, but it is something that seems to touch my heart to be able to help out this way. (daughter)

#### Barriers and related recommendations to improve the implementation of Namaste Care

Our study findings, based on field notes and staff interviews, highlighted perceptions about the barriers and recommendations for improvement when implementing Namaste Care in LTC, in particular areas around staffing, training, timing of program, and adverse events that occurred during the implementation of Namaste Care.

##### Staffing

When the program was first implemented, the LTC homes found it difficult to execute with the current contingent of staff. We originally thought that the program could be achieved with nurses and personal support workers running the program. When we had meetings with the LTC homes, we found that more delineation of roles was needed and that the LTC homes preferred to have personal support workers and recreation staff implement the program. In order to successfully implement the program, we had housekeeping porter residents to and from the Namaste Care room, dietary helped with the tracking and distribution of liquids, and housekeeping was scheduled to help launder residents’ Namaste Care blankets. One reoccurring barrier to Namaste Care that was brought up was staff burden over time. This involved the fact that the program does not require any extra staffing and simply redistribution of staff members. Many staff members, including recreation therapists, identified that they felt they are already overworked and do not have enough time to do any more. The personal support workers/care aides did not like to leave their co-workers on the floor alone while they went to Namaste Care for safety reasons (i.e., transferring some residents requires two people) and fear they were neglecting the other residents in the home.

Being that is a very busy time of the morning, and getting people there you know with the care aides it is hard to get people there in the morning, and we don’t have the staff (recreation therapist)

##### Training staff

As the program was being implemented, we quickly realized that a training program needed to be established for new staff who would be implementing the program. As a result, we developed a staff training toolkit and posted videos of Joyce Simard’s training sessions on the LTC homes online learning portals. In this way, staff were able to access background training materials before shadowing staff in the Namaste Care room.

##### Program time changes

The Namaste Care program was originally designed to run for 4 h a day, 7 days a week. Due to insufficient staffing on the weekends, it was immediately realized that the program could only be run from Monday to Friday. Although the program originally ran for 2 h in the morning and 2 h in the afternoon, this changed to 1.5 h each in the morning and afternoon for one of the sites. The decrease in time was caused by staff shortages. The program’s time was also impacted when it had to shut down due to infection control outbreaks. Program closure was implemented in order to ensure that diseases were not spread between floors or buildings. The time of the program for individual residents was also adapted for individual resident preferences. Some residents preferred to attend only in the afternoon, as they liked to sleep in. Other residents who were prone to bedsores only attended in the morning or afternoon in order to decrease the chance of bedsores.

##### Adverse event

Another barrier or challenge that staff participants described was related to adverse events such as incidence of skin breakdown. This issue was raised at both sites:

well it doesn’t hurt them, the residents. Except there are some that can’t go twice a day because they have skin break down, I mean they just can’t handle it body wise (PSW/CA)

## Discussion

This study demonstrated that implementing the Namaste Care program in Canada was feasible but required sessions to be shortened and some shifting of staff roles to help implement it, which in some cases was very challenging. Our study findings show that, with current staffing levels, implementing Namaste Care the way it was intended (e.g., two 2-h sessions per day) placed a great deal of demand on staff. However, staff and family members highly endorsed Namaste Care, demonstrating its acceptability in LTC. Finally, our study showed positive trends for improved outcomes, such as resident quality of life, and decreased pain and medication use.

Although this study showed that Namaste Care could be implemented within “real-world” conditions with shortened sessions, the feasibility of implementing Namaste Care in LTC could be greatly enhanced with support from others, for example, family members, students, and volunteers. Without the addition of hiring extra staff, our study findings highlighted several conditions that need to be in place to allow this to happen. Based on our initial interviews with LTC staff that occurred before we implemented Namaste Care, staff reported that they should be involved in decision-making to adjust the program launch and have more choice in its design and selection of residents who would be appropriate for it [32, 33]. We found that it is important to work with the individual LTC home to strategize how to implement Namaste Care within the context of their own setting in light of the resources and supports available to them, in hopes that developing a schedule together may improve sustainability. For example, LTC homes may choose to limit the program to weekdays only, shorten the afternoon or morning session, or optimize recreation staff as Namaste carers. In our study, the recommended number of hours per day of implementing Namaste Care was reduced. It is unclear based on these study results and other literature [27] what is the right “dose” of the program to produce positive results. Future work is needed to understand what “dose” is needed to produce effective outcomes, and then, staffing considerations can be considered based on that information.

Most importantly, our study findings highlighted that in order for Namaste Care to be successfully implemented, staff need to make it a priority. To do this, staff need to be supported by the leadership within the LTC home to make it a priority. This may include shifting other tasks assigned to staff to offset the demands of implementing Namaste Care, or prioritizing other resources (e.g., volunteers) to help implement it. If not, Bunn et al. suggest that Namaste Care may require additional resources to implement it, particularly at the initial launch [27]. Anderson et al. [53] found that staff missed sessions to implement a sensory Snoezelen room intervention for people with dementia because staff did not view it as a priority in their work. Clearly, the importance of adequate buy-in from the LTC home is essential to successfully implement Namaste Care.

It was clear that some of the components of Namaste Care were implemented more often than others for residents, such as listening to music, having hands and face washed, being given a massage, or drinking a beverage. These more commonly implemented activities are consistent with those that were identified from a realist review and stakeholder interviews as being the core elements of Namaste Care [27]. A programmatic approach provides structure, guidelines, and permission for staff to provide the kind of care that is needed for residents with advanced dementia. Moreover, Bunn et al. [27] stated that Namaste Care helps staff to manage challenging behaviors using a multisensory approach. In this manner, “moments of connection” are developed between staff and residents to help offset challenging behaviors that often result from anxiety or agitative episodes [27].

Our study findings revealed positive trends in study outcomes with implementing Namaste Care, although only decreased use of antidepressants was statistically significant. Our qualitative findings support the quantitative outcome findings, in that many positive moments were described by staff and family members during Namaste Care. These moments, including residents speaking, being more interactive, smiling, and not seeming depressed or “emotionally hurt” have been reported in other Namaste Care research as well [54]. It is interesting to note the positive trend in increased use of pain medications and decreased pain levels in our sample. Perhaps, the focus within the Namaste Care program on daily pain assessments using a behavioral assessment tool (i.e., PACSLAC-II) contributed to this trend.

Despite these positive trends in our study outcomes, we did observe some adverse events (i.e., skin breakdown) that occurred in our study participants, but we are unsure if these events occurred due to implementing Namaste Care or whether they would have occurred anyways. Still, these adverse events are concerning. In light of these findings, more focused attention is needed to minimize them in future work which could include (a) using special chairs for high-risk residents who may be prone to skin breakdown and (b) regular skin assessments for residents that are included as a component of daily Namaste Care activities.

Our findings demonstrated that Namaste Care contributes to person-centered care for residents with advanced dementia, consistent with other literature [27]. Davies et al. found that families felt it was important that people with dementia continue to participate in activities and be treated as unique individuals despite the fact that dementia has “taken away” the person they once knew [55]. According to Burns et al., providing regular structured access to social and physical stimulation for people with dementia can lead to decreased agitation and improved mood, by developing trust between residents and carers where staff are “intentionally present,” offering a sense of familiarity and reassurance for residents [27]. Moreover, Davies et al. found that family members reported that having nursing staff physically present and verbally communicating with dying residents, even when the resident with dementia could no longer communicate, had inspired their trust and relieved their anxiety [55]. Thus, Namaste Care appears to improve the well-being of both residents and their family members.

Although the qualitative interview findings with family members revealed resounding support for Namaste Care, our quantitative data, one of our feasibility indicators (family visits), showed limited family involvement during the Namaste Care sessions. Qualitative findings showed that Namaste Care reassured family in that they knew the resident was receiving meaningful activity/engagement, which allowed them to “take a break” or some respite time which can be positive. Alternatively, perhaps Namaste Care could provide an opportunity for some meaningful engagement between families and residents, as opposed to situations where family visitation is distant and stressful, resulting in decreased frequency and duration and leaving people with dementia to enjoy limited to no social interaction outside of those provided during the course of routine clinical care [56–58]. Advanced dementia can cause carer stress, anxiety, and decreased well-being [59–63]. Unfortunately, carers can feel a sense of guilt and loss in caregiving after their family member has relocated to a residential or hospital setting as their normal relationship has changed [ 59]. Despite these negative effects, carers can also experience positive aspects including companionship, enjoyment, and reward, when provided opportunity to support and engage in caring as dementia progresses [61]. However, as people with dementia become less able to respond to carers, these positive effects are lost and there is less opportunity for easy communication and shared activities. This contributes to added stress and burden and diminishes the quality of their family visits, leading to further social exclusion and isolation. Despite this, few studies have examined the impact of interventions on carers of persons with advanced dementia [3]. Advanced dementia is a critical time for carer involvement, given that involvement at this time may have an impact on the dying experience of the person with dementia and on the carer’s bereavement and post-bereavement experience [64–67]. Future research is needed to explore ways to support family members to participate in Namaste Care to promote well-being for both parties.

There were some limitations to this study. First, our small sample limited the power to show any statistically significant changes in the outcome measures. However, we have determined confidence intervals which will be used to calculate estimated sample sizes in future study, consistent with a goal of conducting definitive studies. Also, the two homes where Namaste Care was implemented were very enthusiastic about its implementation which likely contributed to our positive findings related to feasibility. It may be less feasible to implement Namaste Care in other homes who have less buy-in and support for the program.

## Conclusions

In summary, this feasibility study provided initial support for implementing Namaste Care in LTC homes who are enthusiastic about the program. Positive findings, including decreased antidepressant use and positive feedback from families and care providers, provide preliminary support for its effectiveness and its potential to improve the quality of living and dying for residents with dementia living in LTC. Future work is needed to explore how to engage and support family members in Namaste Care in meaningful activities with residents. In doing so, efforts may result in both improved quality of living for residents with advanced dementia and their family members who support them.

## Data Availability

All data generated or analyzed during this study are included in this published article. The datasets generated and/or analyzed during the current study are not publicly available due to constraints of our ethical review approvals related to privacy laws.
